# Therapeutic effect of a novel histone deacetylase 6 inhibitor, CKD-L, on collagen-induced arthritis in vivo and regulatory T cells in rheumatoid arthritis in vitro

**DOI:** 10.1186/s13075-017-1357-2

**Published:** 2017-07-03

**Authors:** Bo Ram Oh, Dong-hyeon Suh, Daekwon Bae, Nina Ha, Young Il Choi, Hyun Jung Yoo, Jin Kyun Park, Eun Young Lee, Eun Bong Lee, Yeong Wook Song

**Affiliations:** 1 Department of Molecular Medicine and Biopharmaceutical Sciences, BK 21 plus Graduate School of Convergence Science and Technology, College of Medicine, Seoul National University, Seoul, Korea; 2 Department of Pharmacology and Toxicology, CKD Research Institute, CKD Pharmaceutical Company, Seoul, Korea; 3 Division of Rheumatology, Department of Internal Medicine, Seoul National University College of Medicine, Seoul, Korea

**Keywords:** Histone deacetylase 6, Histone deacetylase inhibitor, Rheumatoid arthritis, Collagen-induced arthritis, Regulatory T cell

## Abstract

**Background:**

Histone deacetylase (HDAC) inhibitor has recently been reported to have a therapeutic effect as an anti-inflammatory agent in collagen-induced arthritis (CIA). We investigated the therapeutic effect of a new selective HDAC6 inhibitor, CKD-L, compared to ITF 2357 or Tubastatin A on CIA and regulatory T (Treg) cells in patients with rheumatoid arthritis (RA).

**Methods:**

CIA was induced by bovine type II collagen (CII) in DBA/1 J mice. Mice were treated with HDAC inhibitor for 18 days. Arthritis score was assessed and histological analysis was performed by hematoxylin and eosin (H&E) stain. Cytotoxic T-lymphocyte associated protein (CTLA)-4 expression in induced Treg cells was analyzed and suppression assay was analyzed using Treg cells and effector T (Teff) cells isolated from naive C57BL/6 mice by flow cytometry. Cytokines were analyzed in peripheral blood mononuclear cells (PBMC) of five patients with RA by enzyme-linked immunosorbent assay (ELISA) and real-time polymerase chain reaction (PCR). Tumor necrosis factor (TNF) was analyzed using PMA- activated THP-1 cells by ELISA. Suppression assay was analyzed using Treg cells and Teff cells isolated from RA patients by flow cytometry.

**Results:**

In the CIA model, CKD-L and Tubastatin A significantly decreased the arthritis score. CKD-L increased CTLA-4 expression in Foxp3^+^ T cells and inhibited the proliferation of Teff cells in the suppression assay. In RA PBMC, CKD-L significantly inhibited TNF and interleukin (IL)-1β, and increased IL-10. CKD-L and Tubastatin A inhibited TNF secretion from PMA-activated THP-1 cells. CKD-L and ITF 2357 inhibited the proliferation of Teff cells in RA patients in the suppression assay. Tubastatin A had no effect on inhibition of proliferation.

**Conclusion:**

CKD-L decreased the arthritis score in CIA, reduced the expression of TNF and IL-1β, and increased the expression of IL-10 in PBMC from RA patients. CKD-L increased CTLA-4 expression and the suppressive function of Treg cells. These results suggest that CKD-L may have a beneficial effect in the treatment of RA.

## Background

Histone deacetylase (HDAC) and histone acetyltransferase (HAT) play important roles in the regulation of gene transcription [1]. The positively charged lysine in the N-terminal tail of histones is neutralized by acetylation of histone by HAT, and the binding affinity between the DNA backbone and histones is decreased because histones do not bind to the negatively charged phosphate groups in DNA. The decreased level of interaction between DNA and histones increases gene transcription by promoting the binding of transcription factors to DNA [2–4].

In opposition to HAT, deacetylation of histone by HDAC represses gene transcription through chromatin condensation [5]. HDAC is classified into four classes based on DNA sequence similarity and function. Class I, II, and IV HDACs are classical HDACs that have a zinc-dependent active site, whereas class III HDACs are a family of nicotinamide adenine dinucleotide (NAD^+^)-dependent proteins. Class I HDACs (HDACs 1–3 and 8) are primarily found in the nucleus, and class II HDACs (HDAC 4–7, 9, and 10) are found in both the nucleus and the cytoplasm and can shuttle between the two. The class IV HDAC (HDAC11) shares structural similarities with both class I and class II HDACs. Class III HDACs (SirT 1–7) have distinct structures and different mechanisms of action. Their enzymatic activity depends on the cofactor NAD^+^ [1, 6, 7].

HDAC6 is primarily located in the cytoplasm and has a unique structure that contains two homologous catalytic domains and an ubiquitin binding domain at the C-terminal end [8, 9]. HDAC6 has been reported to involve many important biological processes, including cell migration, immune response, viral infection, and the degradation of misfolded proteins. The substrates of HDAC6 are α-tubulin, heat shock protein (HSP) 90, peroxiredoxin (PRX), and cortactin, and HDAC6 regulates acetylation of multiple proteins by forming various complexes with other partner proteins. These diverse functions of HDAC6 offer potential therapeutic targets in various diseases such as systemic lupus erythematosus, cancer, and diabetes [10, 11]. The inhibition of HDAC6 was reported to enhance the suppressive activity of regulatory T cells (Treg cells) in inflammation and autoimmunity [12].

Histone deacetylase inhibitors modulate the function of HDACs and activate and/or repress gene expression. HDAC inhibition leads to cell cycle arrest, cell growth, cell differentiation, and apoptotic death of transformed cells [13, 14]. HDAC inhibitors have been developed; the pan HDAC inhibitors, such as ITF 2357 and SAHA, inhibit all HDACs, and the selective HDAC inhibitors, such as Tubastatin A and Tubacin, inhibit HDAC6 specifically [15].

Epigenetic regulation plays an important role in inflammatory autoimmune diseases including rheumatoid arthritis (RA) through changes in histone modification [16]. RA is a chronic autoimmune disease characterized by inflammatory synovitis and progressive destruction of joint cartilage and bone, leading to swelling, pain, stiffness, and loss of function [17, 18]. The etiology of RA remains unclear, but genetic background and environmental factors play important roles in the disease [19].

Current medical treatments for RA include nonsteroidal anti-inflammatory drugs (NSAIDs) and disease-modifying antirheumatic drugs (DMARDs), including methotrexate (MTX), sulfasalazine, and tumor necrosis factor (TNF) inhibitors (infliximab, etanercept, and adalimumab) [20]. TNF is a proinflammatory cytokine that is associated with the development of RA. TNF blockade can be an effective treatment for RA [21]. However, about 40% of RA patients do not respond to antiTNF therapy, so novel therapies are required for patients who show no response [1, 5].

HDAC inhibitors have been reported to have potential therapeutic effects as anti-inflammatory agents in many studies including those on collagen-induced arthritis (CIA) [5, 22–25]. HDAC inhibitors suppressed the production of interleukin (IL)-6 in RA fibroblast-like synoviocytes (FLS) and macrophages by promoting mRNA decay [26]. In RA, macrophages and T cells are major sources of proinflammatory cytokines [27]. The activation, survival, and apoptosis of macrophages are regulated by acetylation and deacetylation of histones. HDAC inhibitors suppress TNF and IL-6 production and cytokine gene transcription and induce apoptosis in macrophages [28]. HDAC inhibitors reduce the secretion of proinflammatory cytokines, such as TNF and IL-6, in peripheral blood mononuclear cells (PBMC) of RA patients and reduce the secretion of TNF, IL-1α, IL-1β, and interferon (IFN)-γ in lipopolysaccharide (LPS)-stimulated normal PBMC [1, 24]. Treg cells are a subset of CD4^+^ T cells that have an immunosuppressive role in immune tolerance [29]. Treg cells from RA patients are defective in suppressing proinflammatory cytokine production [30].

Tubastatin A inhibited HDAC6 and induced Foxp3 expression, resulting in enhanced Treg function [12]. In addition, Tubastatin A suppressed TNF and IL-6 from the THP1 macrophage cell line and inhibited CIA [31]. However, it is still unclear whether HDAC6-selective inhibitors have a similar immunoregulatory function in RA patients.

Here, we introduce a new HDAC6 selective inhibitor, CKD-L, developed by the Chong Kun Dang Pharmaceutical Corporation. Although the tertiary amine tricycle of Tubastatin A has excellent HDAC6-selective inhibitory activity, the poor PK profile and genotoxicity of Tubastatin A significantly limited its development for clinical use. CKD-L has a bicycle instead of a tertiary amine tricycle, resulting in better drug-like structure and profiles without any genotoxicity in the Ames test and the chromosome aberration test.

We investigated the therapeutic effect of the novel selective HDAC6 inhibitor, CKD-L, and compared its effect to those of the pan HDAC inhibitor, ITF 2357, and the selective HDAC6 inhibitor, Tubastatin A, in CIA, PBMC, and Treg cells from patients with RA. CKD-L showed comparable efficacy to Tubastatin A in the CIA model. Moreover, CKD-L showed immunomodulatory effect in RA patients, such as improvement of Treg function and suppression of TNF production in PBMC suggesting that HDAC6 may be an important therapeutic target protein to control RA, and that HDAC6-specific inhibitors such as CKD-L can be developed for clinical use.

## Methods

### Chemicals

CKD-L is a new histone deacetylase 6 inhibitor developed by the Chong Kun Dang Pharmaceutical Corporation (CKD Pharm). ITF 2357 and Tubastatin A were also provided by CKD Pharm and were used as positive controls (pan HDAC inhibitor and selective HDAC6 inhibitor, respectively). These chemicals were dissolved in dimethyl sulfoxide (DMSO; Sigma-Aldrich, St. Louis, MO, USA). The stock solutions were stored at –20 °C and were diluted to the required concentrations in growth media as necessary. Vehicle (DMSO) was used in the negative control groups.

### Animals

DBA/1 J mice (male, 6 weeks old) and C57BL/6 mice (male, 6 weeks old) were purchased from Central Lab Animal, Inc. (SLP, Hamamatsu, Japan). The animals were kept in a specific pathogen-free facility. The animals were provided with standard diet and water ad libitum. The animal study was reviewed and approved by the CKD Institutional Animal Care and Use Committee (approval number: S-16-038).

### CIA experiments

Before the first immunization, bovine type II collagen (CII; Chondrex, Redmond, WA, USA) 2 mg/ml in 0.05 M acetic acid and an equal volume of complete Freund’s adjuvant (CFA; Chondrex) were mixed by using a precooled homogenizer in an ice bath; 100 μl of the emulsion was injected intradermally at the base of the tail of each DBA1/J mouse on day 1.

On day 21 after the first immunization, the mice were boosted with the second immunization. Bovine type II collagen (2 mg/ml in 0.05 M acetic acid) and an equal volume of incomplete Freund’s adjuvant (IFA; Chondrex) were mixed by using a precooled homogenizer in an ice bath; 100 μl of the emulsion was injected intradermally at the base of the tail of each mouse.

#### Drug administration and evaluation of arthritis

After the second immunization, the mice were divided into four equal groups on the basis of their body weight and given either vehicle or HDAC6 inhibitors. CKD-L and Tubastatin A were dissolved in cremophor EL:ethanol:saline (1.5:1.5:7). Mice were treated with vehicle (*n* = 10), CKD-L (15 or 30 mg/kg, *n* = 10 for both), or Tubastatin A (30 mg/kg, *n* = 9) by subcutaneous injection every day for 18 days. Arthritis score and body weight were assessed every 2 days after the first drug administration. On day 38, all mice were sacrificed after anesthetization for pathological analysis.

#### Arthritis score assessment

Arthritis score was evaluated as 5 scales as previously reported [32]: 0 = no evidence of erythema and swelling; 1 = erythema and mild swelling confined to the tarsals or ankle joint; 2 = erythema and mild swelling extending from the ankle to the tarsals; 3 = erythema and moderate swelling extending from the ankle to metatarsal joints; 4 = erythema and severe swelling encompass the ankle, foot and digits, or ankylosis of the limb.

Arthritis was scored using a scale of 0–4 for each paw. The total score per mouse was summed from 0 to 16 (normal = 0; maximum severe score = 16).

#### Body weight change after onset of arthritis

Change in body weight (%) is a general marker of various diseases including RA. Thus, body weight change after the onset of arthritis can reflect disease progression.

#### Histological score

The knee joints and hind paws of mice in each group were dissected for histopathological analysis. Dissected knee joints and hind paws were fixed in 10% phosphate-buffered formalin (pH 7.4), decalcified in 10% ethylenediaminetetraacetic acid (EDTA) for approximately 2 weeks, embedded in paraffin, and then processed as per standard methods. The sections were stained with hematoxylin and eosin (H&E) and scored by seven independent, blinded observers. Synovial inflammation, bone erosion, cartilage damage, and leukocyte infiltration were assessed using a scale of 0–4 (normal = 0; mild = 1; moderate = 2; marked = 3; severe = 4). Histological score was calculated by summation of these scales (normal = 0; maximum score = 16).

### CTLA-4 expression using induced murine Treg cells

#### Cell preparation and enzymatic digestion

Spleens from naive C57BL/6 mice were harvested and then incubated with 2 mg/ml collagenase D (Roche, Mannheim, Germany) for 30 min at 37 °C. Spleens were meshed through a 70-μm cell strainer (Becton Dickinson, Franklin Lakes, NJ, USA) to obtain single cell suspensions.

Treg cells (CD4^+^CD25^+^ T cells) and effector T (Teff) cells (CD4^+^CD25^–^ T cells) were isolated from splenocytes using a CD4^+^CD25^+^ regulatory T Cell Isolation Kit (Miltenyi Biotec, Bergisch-Gladbach, Germany) by magnetic cell sorting (MACS) separator according to the manufacturer’s instructions. The purity of each cell population (>95%) was confirmed by flow cytometry.

#### Treg cell induction and drug treatment

CD4^+^CD25^–^ T cells were isolated from splenocytes using the CD4^+^CD25^+^ regulatory T Cell Isolation Kit (Miltenyi Biotec) as a negative fraction by MACS separator. The purity of the isolated cell population (>95%) was confirmed by flow cytometry.

CD4^+^CD25^–^ T cells (5 × 10^5^ cells/ml/well) were incubated with vehicle or HDAC6 inhibitor (1 to 10 μM) in the presence of antiCD3/CD28 beads (T cell Activation/Expansion kit, Miltenyi Biotec), and recombinant mouse transforming growth factor (TGF)-β2 (40 pg/ml; R&D systems Inc., Minneapolis, MN, USA) in a 48-well plate (Becton Dickinson) for 6 days at 37 °C in a humidified 5% CO_2_ incubator.

For surface staining, cells were incubated with PE-Cy7-labeled antimouse CD4 (eBioscience), and APC-labeled antimouse CD25 (eBioscience) for 20 min at room temperature (RT). For intracellular staining, the cells were fixed and permeabilized with fix/permeabilization buffer (eBioscience) for 20 min at 4 °C, washed twice with wash buffer, and then incubated with AlexaFluor488-labeled antimouse forkhead box P3 (Foxp3) and PE-labeled antimouse CTLA-4 (eBioscience) for 20 min at 4 °C in the dark. CTLA-4 expression of induced Foxp3^+^ Treg cells was analyzed by FACS CantoII flow cytometry (BD bioscience, San Jose, CA, USA) and the results were analyzed with FlowJo software (TreeStar Inc. Ashland, OR, USA).

### Suppression assay using murine natural Treg cells and Teff cells

#### Isolation of Treg cells and Teff cells

Treg cells and Teff cells were isolated from splenocytes of naive C57BL/6 using CD4^+^CD25^+^ regulatory T Cell Isolation Kit (Miltenyi Biotec) by MACS separator according to the manufacturer’s instructions. The purity of each cell population was >95% as confirmed by flow cytometry.

#### Labeling of Teff cells

Teff cells were labeled with 5 uM Cell Proliferation Dye eFluor®670 (eBioscience) for 10 min at 37 °C under protection from light.

#### Suppression assay

Treg cells were mixed with eFluor®670-labeled Teff cells at ratios of 4:1 and 0:1 in RPMI1640 media (Gibco, MA, USA) containing 10% heat-inactivated fetal bovine serum (FBS; Gibco), 1% penicillin/streptomycin (P/S; Gibco), 1 mM sodium pyruvate (Gibco), and 1% 2-mercaptoethanol (Gibco) in a 96-well round bottom plate (Becton Dickinson). The mixed cells were incubated with vehicle (DMSO final concentration 0.1%) or HDAC6 inhibitor (1 to 10 uM) in the presence of antiCD3/CD28 beads (T cell Activation/Expansion kit, Miltenyi Biotec) for 4 days. Proliferation of Teff cells was analyzed using FACS LSR Fortessa (BD bioscience). The results were analyzed by FlowJo software (TreeStar Inc.).

### RA patients

#### RA patients and healthy controls

Patients with RA were enrolled at the Rheumatology Clinic, Seoul National University Hospital. All RA patients fulfilled the 1987 American College of Rheumatology (ACR) classification criteria of RA [33]. Healthy controls were volunteers who did not have RA. This study was approved by the Institutional Review Board (IRB No.: 1207-153-421) and informed consent was obtained from each patient.

#### Methylthiazol tetrazolium (MTT) assay and cell viability

Heparinized blood of RA patients was diluted with an equal volume of phosphate-buffered saline (PBS), and diluted whole blood was carefully layered over Ficoll-Paque™ PLUS (specific gravity 1.007 g/ml; GE Healthcare Life Science, Uppsala, Sweden) (blood:PBS:Ficoll = 1:1:1). After centrifugation at 1700 rpm for 30 min at RT, PBMC were isolated from the plasma and Ficoll interface and washed twice with PBS.

Isolated PBMC were cultured in a 96-well plate and treated with LPS (100 ng/ml; Sigma-Aldrich) and vehicle or HDAC inhibitor (0.01 to 5 μM) in RPMI 1640 media containing 10% (v/v) FBS and 1% P/S (10,000 units/ml; Gibco/BRL, Grand Island, NY, USA) for 24 h at 37 °C in a humidified 5% CO_2_ incubator. For the MTT assay, 10 μl CCK-8 solution (CCK-8; Dojindo Laboratory, Kumamoto, Japan) was added to each well and incubated for 2 h at 37 °C in the dark. The absorbance in solution was measured by a Luminex 200 (Luminex Corporation, Austin, TX, USA) at 450 nm.

#### Cytokine assay

Isolated PBMC were cultured in a 48-well plate (Becton Dickinson) and treated with LPS (100 ng/ml) and vehicle or HDAC inhibitor (0.01 to 5 μM) in RPMI 1640 media containing 10% FBS and 1% P/S for 24 h at 37 °C in a humidified 5% CO_2_ incubator. Cell culture supernatant was harvested for multiplex immunoassay of TNF, IL-1β, and IL-10 using Bio-Plex Pro™ Assay (Bio-Rad Laboratories, Hercules, CA, USA) according to the manufacturer’s instructions. The secretion of IL-6 in the cell culture supernatant was measured by using the Human IL-6 DuoSet enzyme-linked immunosorbent assay (ELISA) kit (R&D) according to the manufacturer’s instructions.

#### RNA preparation

Cells were harvested and washed with PBS for RNA preparation. Total RNA was extracted using an RNeasy micro kit (Qiagen, Valencia, CA, USA) according to the manufacturer’s instructions. In brief, 350 μl RLT buffer was added to the pelleted cells (5 × 10^5^ cells) to lyse them. An equal volume of 70% ethanol was added to the lysate and gently mixed by pipetting. The mixed lysate was transferred into an RNeasy MinElute spin column placed in a 2 ml collection tube and centrifuged at 8000 g for 15 s at RT. After washing with 350 μl RW1 buffer, 80 μl DNase I working solution was added directly to the column membrane and incubated for 15 min at RT. After washing with 350 μl RW1 buffer, 500 μl RPE buffer was added to the column, which was centrifuged at 8000 g for 15 s at RT. Then 500 μl 80% ethanol was added to the column and centrifuged at 8000 g for 2 min at RT. The column was transferred in a new 1.5-ml collection tube and 14 μl RNase-free water was directly added to the center of the column membrane. The column was centrifuged at full speed for 1 min at RT to elute the RNA. The concentration of RNA was determined by measuring absorbance at 260 nm with a Nanodrop ND-100 spectrometer (Nanodrop Technologies, Wilmington, DE, USA).

#### Reverse transcription-polymerase chain reaction (RT-PCR) and complementary DNA (cDNA) synthesis

First-strand cDNA was synthesized from an equal concentration of total RNA by using the SuperScript® III First-Strand Synthesis System (Invitrogen, Carlsbad, CA, USA). The reaction was conducted in a final volume of 10 μl containing 20 ng total RNA, 5 μM oligo (dT)_20_ primer, and 1 mM deoxynucleotide triphosphate (dNTP) mixture in a 200-μl tube. The tube was incubated for 5 min at 65 °C and placed on ice for at least 1 min. A cDNA synthesis mix solution was prepared with 10× RT buffer, 10 mM DTT, 50 mM MgCl_2,_ 2 units of RNaseOUT, and 10 units of SuperScript® III RT. Then 10 μl of that mix solution was added to each reaction mixture, mixed gently, and incubated for 50 min at 50 °C and for 5 min at 85 °C, and then placed on ice. To remove the remaining RNA, 1 μl RNase H was added to each reaction mixture for 20 min at 37 °C before RT-PCR was performed with a PTC 200 thermal cycler (MJ research Inc., Waltham, MA, USA).

#### cDNA pre-amplification

cDNA was amplified using a Taqman PreAmp Master Mix (Applied Biosystems, Foster City, CA, USA) according to the manufacturer’s instructions. In brief, 10 μl 20× Taqman gene expression assay was added to a tube and Tris-EDTA buffer was added to 1 ml total volume. The reaction was performed in a final volume of 50 μl containing 12.5 μl synthesized cDNA, 5 μl 2× Taqman preamp master mix, 12.5 μl 0.2× pooled assay mix, and 20 μl nuclease-free water. The reactions were incubated for 10 min at 95 °C for one cycle, and then for 15 s at 95 °C and 4 min at 65 °C for 10 cycles. The amplified products were used after being diluted with Tris-EDTA buffer at a ratio of 1:5 before real-time PCR.

#### Quantitative real-time PCR

Quantitative real-time PCR was performed by running a Taqman probe in an ABI 7500 real-time PCR system (Applied Biosystems, Foster Ctiy, CA, USA) according to the manufacturer’s instructions. Human IL-10 (Hs00961622), TNF (Hs01113624), and 18S (Hs99999901)-specific probes were purchased from Applied Biosystems.

The reaction was performed in a final volume of 20 μl containing 5 μl diluted amplified cDNA, 1 μl 20× Taqman gene expression assay buffer, 10 μl 2× Taqman gene expression master mix, and 4 μl nuclease-free water. The reactions were incubated for 15 min at 94 °C for one cycle and then at 94 °C (15 s), 59 °C (30 s), and 72 °C (30 s) for 40 cycles. The quantity of target gene was calculated by the difference between the target gene and reference gene in the threshold cycle (Ct). Relative gene expression was determined using the 2^–ΔΔCt^ method by normalizing with 18S as a reference gene.

### TNF secretion in phorbol 12-myristate 13-acetate (PMA)-activated THP-1 cell

THP-1 cell is a human monocytic cell line, and PMA-activated THP-1 releases the inflammatory cytokine TNF. THP-1 cells were cultured in a 24-well plate (1 × 10^5^ cells/well) and activated with PMA (10 ng/ml; Sigma-Aldrich) in RPMI 1640 media containing 10% FBS and 1% P/S for 24 h to induce macrophage differentiation. Cells were washed with PBS, treated with vehicle or HDAC inhibitor (0.1 to 10 μM) for 24 h, and then treated with 100 ng/ml LPS for 4 h in a humidified 5% CO_2_ incubator. The cell culture supernatant was harvested and TNF secretion was measured using a TNF ELISA kit (eBioscience) according to the manufacturer’s instructions.

### Induction of Treg cells from PBMC of RA patients

#### Isolation of CD4^+^CD25^–^ T cells

CD4^+^CD25^–^ T cells were isolated from PBMC of RA patients by MACS separator according to the manufacturer’s instructions. In brief, PBMC were incubated with a cocktail of biotin-conjugated monoclonal antihuman antibodies: CD8, CD14, CD15, CD16, CD19, CD36, CD56, CD123, TCRγ/δ, and CD235a (Glycophorin A) in MACS buffer (PBS, 0.5% bovine serum albumin (BSA), and 2 mM EDTA; pH 7.2) for 10 min at 4 °C in the dark. Then, cells were incubated with antibiotin microbead for 15 min at 4 °C. Non-CD4^+^ cells were indirectly magnetically labeled using a cocktail of biotin-conjugated antibodies as primary reagent and antibiotin monoclonal antibodies conjugated to microbead as secondary reagent. The labeled cells were removed as a negative fraction using an LD column. Isolated CD4^+^ cells were incubated with microbead conjugated to a monoclonal antihuman CD25 antibody (MACS®; Miltenyi Biotec) for 15 min at 4 °C. The labeled cells were removed as a negative fraction using an LD column. The purity of isolated CD4^+^CD25^–^ T cells was >95% as confirmed by flow cytometry.

#### Induction of Treg cells

Antihuman CD3 antibody (eBioscience; 200 μl; 2 μg/ml PBS) was added to each well of a 48-well plate. After being sealed, the plate was incubated overnight at 4 °C. The coated plate was washed twice with PBS before seeding. CD4^+^CD25^–^ T cells were cultured with 2 μg/ml antihuman CD28 antibody (BD Pharmingen, San Diego, CA, USA), 2 ng/ml IL-2 (Peprotech, London, UK), 5 ng/ml TGF-β (Peprotech, London, UK), and 20 nM 1,25-dihydroxyvitamin D_3_ (Sigma-Aldrich) in X-VIVO™ 15 media (Lonza, Walkersville, MD, USA) containing 1% P/S and 10% heat-inactivated human serum AB (Lonza) for 5 days at 37 °C in a 5% CO_2_ incubator. Induced Treg cells were harvested and washed with PBS. Cells were cultured in X-VIVO™ 15 media containing 20 ng/ml IL-2 in a new 48-well plate for 2 days. The phenotype of induced Treg cells was confirmed by flow cytometry using PerCP-labeled antihuman CD4 antibody, PE-labeled antihuman CD25 antibody, APC-labeled antihuman Foxp3 antibody, and PE-Cy5-labeled antihuman CTLA-4 antibody.

#### Suppression assay

Teff cells (CD4^+^CD25^–^ T cells) were isolated from PBMC of heathy volunteers by MACS separator and labeled with a CellTrace™ carboxyfluorescein succinimidyl ester (CFSE) cell proliferation kit (Celltrace, Molecular Probes, Invitrogen) according to the manufacturer’s instructions. In brief, isolated Teff cells were resuspended in prewarmed PBS containing 0.1% BSA (Sigma-Aldrich) at a final concentration of 1 × 10^6^ cells/ml. Teff cells were labeled by CFSE solution at 5 mM of the final working concentration for 10 min at 37 °C in the dark. CFSE-labeled Teff cells were added to 5× volume of cold culture media and placed on ice for 5 min to stop the labeling process. Induced Treg cells were mixed with CFSE-labeled Teff cells at ratios of 1:1, 0.3:1, and 0:1 to produce a total cell count per well of 5 × 10^4^/200 μl RPMI 1640 media containing 1% P/S and 10% FBS in a 96-well plate. The mixed cells were incubated with vehicle or HDAC inhibitor (0.01 to 5 μM) in the presence of Dynabeads® human T-activator CD3/C28 (one bead to four cells) for 3 days in the dark in a humidified 5% CO_2_ incubator. Proliferation of Teff cells was analyzed by CFSE dilution using LSR II flow cytometry (BD Bioscience). The results were analyzed with FlowJo software. The suppression ratio was calculated using the fold inhibition of cell proliferation by HDACi vs vehicle when the ratio of Treg:Teff = 1:1.

### Statistical analysis

Statistical analysis was carried out with GraphPad Prism 5 (GraphPad Software Inc., San Diego, CA, USA). All data are expressed as mean ± standard error of the mean (SEM). One-way analysis of variance (ANOVA) followed by Dunnett’s multiple comparison tests and Mann-Whitney *U* tests were used to compare differences between groups. A *p* value <0.05 was considered statistically significant.

## Results

**Fig. 1:**
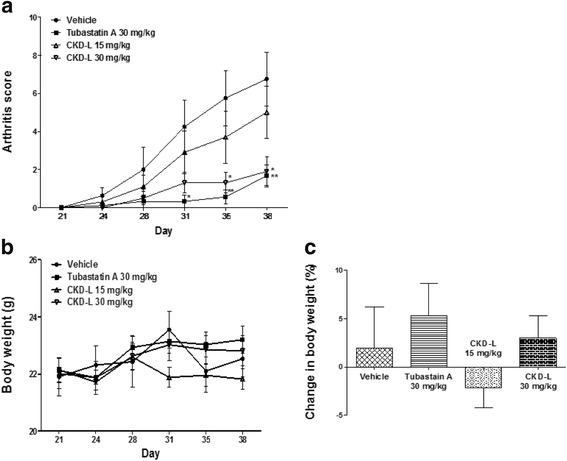
Therapeutic effects of CKD-L on CIA mice. After the onset of arthritis, mice were treated with vehicle, Tubastatin A (30 mg/kg), or CKD-L (15 or 30 mg/kg) subcutaneously every day for 18 days after the second immunization. CKD-L (30 mg/kg) and Tubastatin A significantly decreased arthritis score compared to vehicle (**a**). Body weight did not change during drug administration (**b**). The changes in body weight between day 21 and day 38 was not significantly different among the groups (**c**). *p < 0.05, **p < 0.01, vs vehicle

We assessed the therapeutic effects of CKD-L on the severity of CIA in DBA1/J mice. After the onset of CIA, HDAC inhibitors were administered by subcutaneous injection. Arthritis progressed rapidly in the group treated with vehicle. CKD-L (30 mg/kg) significantly decreased the severity of arthritis compared with vehicle (*p* < 0.05), and Tubastatin A had a similar effect (Fig. 1a). The arthritis scores of the mice treated with 30 mg/kg CKD-L tended to be lower than those of the mice treated with 15 mg/kg CKD-L. Body weight was assessed every 2 days after the first drug administration. The body weights of the animals did not change significantly after administration of HDAC inhibitor (Fig. 1b). The changes in body weight between the first (day 21) and the last (day 38) assessment were not significantly different among the groups (Fig. 1c).

**Fig. 2:**
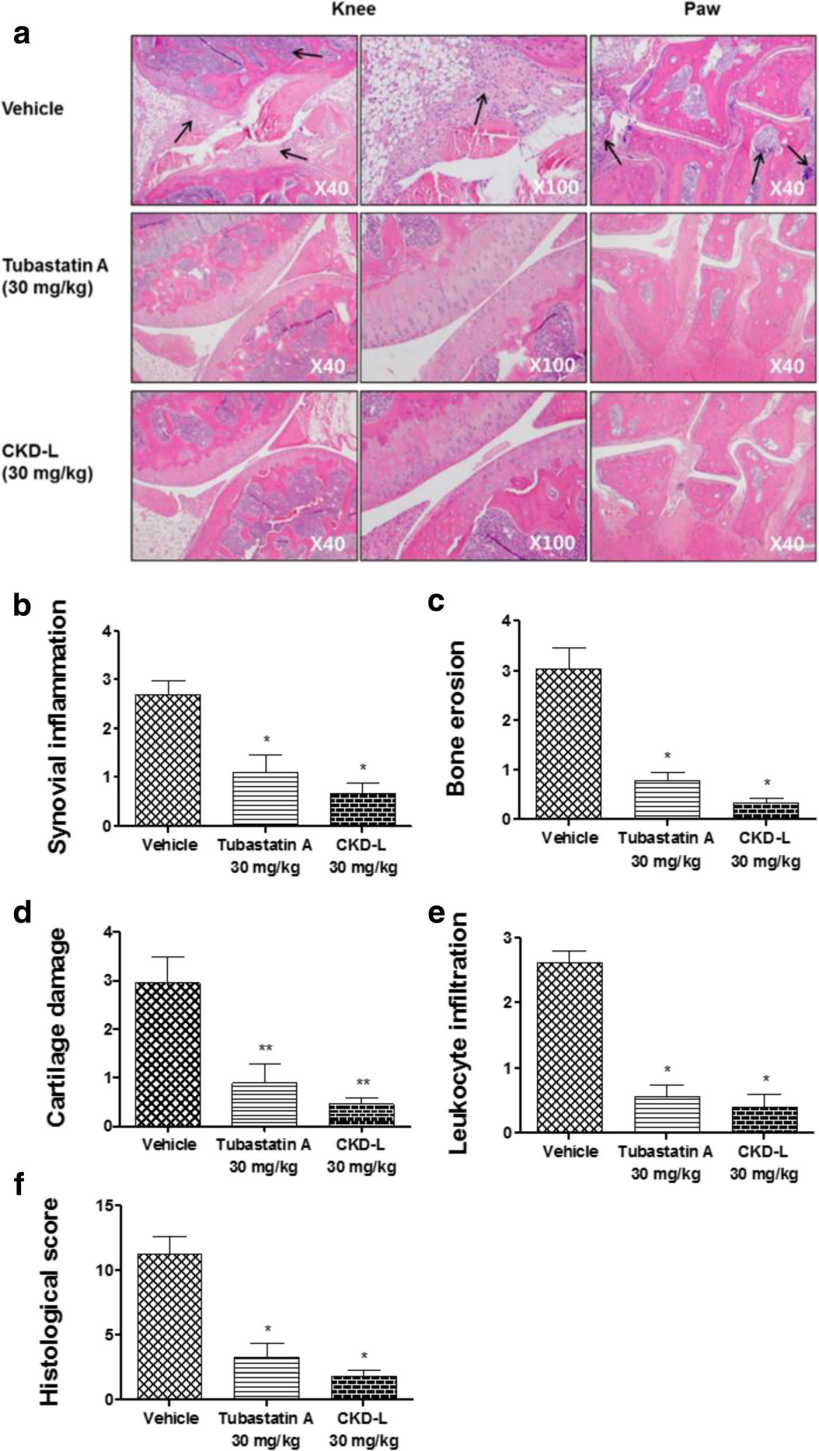
Histological analysis of CIA treated with CKD-L or Tubastatin A. Histological analyses were performed on knee joints (×40, ×100) and hind paws (×40) stained by H&E. Synovial inflammation, bone erosion, cartilage damage, and leukocyte infiltration were observed in the knee joints and hind paws of mice treated with vehicle (*arrows*) compared to Tubastatin A or CKD-L (**a**). The mean score of synovial inflammation (**b**), bone erosion (**c**), cartilage damage (**d**), and leukocyte infiltration (**e**) were calculated using a scale of 0–4 (normal = 0; mild = 1; moderate = 2; marked = 3; severe = 4), and these parameters significantly decreased after CKD-L or Tubastatin A treatment compared to vehicle treatment. Histological score was calculated by summation of these parameters (**f**). Histological score significantly decreased following treatment with CKD-L or Tubastatin A. *p < 0.001, **p < 0.01, vs vehicle

RA is characterized by synovial inflammation, bone erosion, cartilage damage, and leukocyte infiltration in the joints. To investigate the protective effects of CKD-L in the joints, the histological score was assessed in the knees and hind paws of mice by H&E staining. Significantly more synovial inflammation, bone erosion, cartilage damage, and leukocyte infiltration were observed in the knee joints and hind paws of the mice treated with vehicle (arrows in Fig. 2) compared to Tubastatin A or CKD-L (Fig. 2a). CKD-L effectively inhibited arthritis. Synovial inflammation (Fig. 2b), bone erosion (Fig. 2c), cartilage damage (Fig. 2d), and leukocyte infiltration (Fig. 2e) were significantly decreased in the groups treated with CKD-L or Tubastatin A compared to the vehicle-treated group. The histological score was calculated by summation of four parameters: synovial inflammation, bone erosion, cartilage damage, and leukocyte infiltration (Fig. 2f). The histological score was significantly lower in mice treated with CKD-L (*p* < 0.001) or Tubastatin A (*p* < 0.001). These findings suggest that CKD-L may be significantly effective for the treatment of CIA. There was no significant difference in histological score between Tubastatin A and CKD-L.

**Fig. 3:**
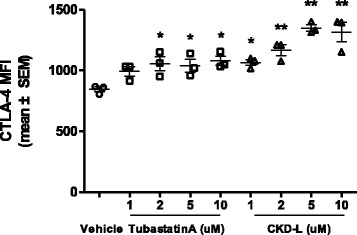
Increased cytotoxic T-lymphocyte antigen-4 (*CTLA-4*) expression by CKD-L in Treg cells from C57BL/6 mice (*n* = 3). CD4^+^CD25^–^ T cells were isolated from splenocytes of C57BL/6 mice. CD4^+^CD25^–^ T cells were incubated with vehicle or HDAC6 inhibitors (1 to 10 μM) in the presence of antiCD3/CD28 beads and recombinant TGF-β2 in a 48-well plate for 6 days. CTLA-4 expression in CD4^+^CD25^+^Foxp3^+^ T cells was analyzed by mean fluorescence intensity (*MFI*; mean ± SEM) using flow cytometry. *p < 0.05, **p < 0.001, vs vehicle

Treg cells have an immunosuppressive role [29]. It was reported that the suppressive function of Treg cells in RA patients is defective [30, 34]. We assessed the effect of CKD-L on Treg cells during their induction. CD4^+^CD25^–^ T cells were isolated from splenocytes of C57BL/6 mice. CD4^+^CD25^–^ T cells were incubated with vehicle or HDAC6 inhibitor (1 to 10 μM) in the presence of antiCD3/CD28 beads and recombinant TGF-β2 in a 48-well plate for 6 days. CTLA-4 expression in CD4^+^CD25^+^ Foxp3^+^ T cells was significantly increased after treatment with CKD-L (*p* < 0.001) or Tubastatin A (*p* < 0.05) compared to vehicle (Fig. 3).

**Fig. 4:**
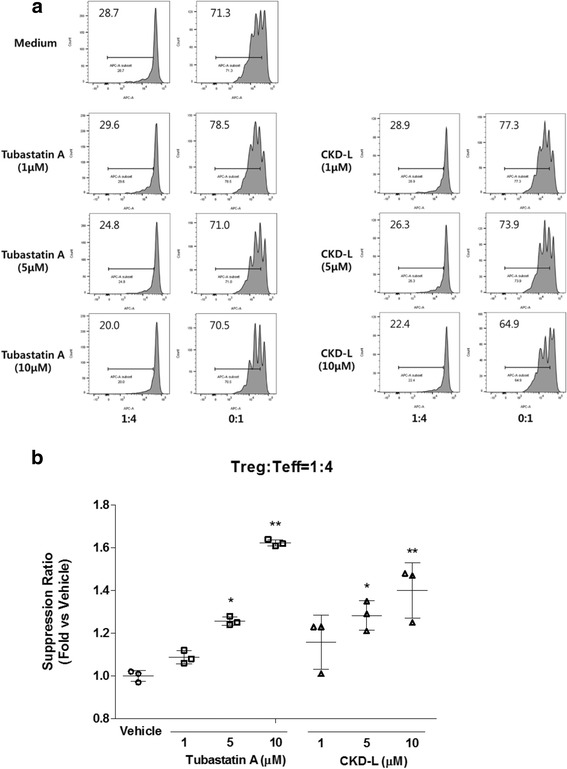
Effect of CKD-L in the suppression assay using regulatory T (*Treg*) cells and effector T (*Teff*) cells from C57BL/6 mice (*n* = 3). Treg cells were mixed with eFluor®670-labeled Teff cells at ratios of 4:1 and 0:1. The mixed cells were incubated with vehicle or HDAC6 inhibitor (1 to10 μM) in the presence of antiCD3/CD28 beads for 4 days. **a** The proliferation of Teff cells was assessed by eFluor®670 dilution using flow cytometry (representative data). **b** Suppression ratio (fold inhibition of cell proliferation by HDACi vs. vehicle) at a ratio of Treg:Teff = 1:4. *p < 0.01, **p < 0.001, vs vehicle

Treg cells have an immunosuppressive role in immune tolerance [29]. It is known that the suppressive function of Treg cells from RA patients was defective in many studies [30, 34]. We assessed the effect of CKD-L on the function of Treg cells. Treg cells (CD4^+^CD25^+^ T cells) and Teff cells (CD4^+^CD25^–^ T cells) were isolated from splenocytes of C57BL/6 mice. Treg cells were mixed with eFluor®670-labeled Teff cells at ratios of 4:1 and 0:1. The mixed cells were incubated with vehicle or HDAC6 inhibitor (1 to 10 μM) in the present of antiCD3/CD28 beads for 4 days. Proliferation of Teff cells was inhibited after treatment with CKD-L and Tubastatin A in a dose-dependent manner compared to vehicle (Fig. 4a). As shown by suppression ratio (fold versus vehicle) at 1:4 ratio of Treg:Teff, the suppression ratio was increased 1.4 times by CKD-L treatment and 1.6 times by Tubastatin A treatment compared to vehicle treatment (*p* < 0.001, respectively) (Fig. 4b).

**Fig. 5:**
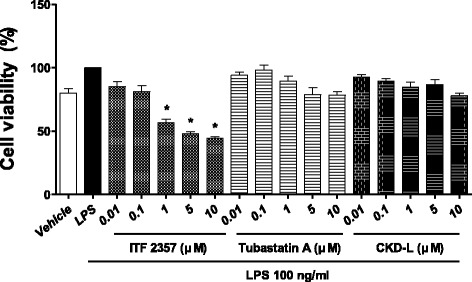
Effects of CKD-L on cell viability in PBMC from RA patients (*n* = 6). PBMC from RA patients were cultured in a 96-well plate for 24 h in the presence of lipopolysaccharide (*LPS*; 100 ng/ml) and HDAC inhibitors at different concentrations (0.01 to 5 μM). Cell viability was determined using the MTT assay. *Bars* represent means and SDs. All experiments were carried out in triplicate. *p < 0.001, vs LPS

To investigate the effect of CKD-L on PBMC of RA patients, cell viability was determined using the MTT assay. Cell viability was not affected by high concentrations of CKD-L or Tubastatin A (Fig. 5). However, ITF 2357 decreased cell viability at concentrations above 1 μM. The HDAC inhibitors were used in the experiments at concentrations that would not affect cell viability.

**Fig. 6:**
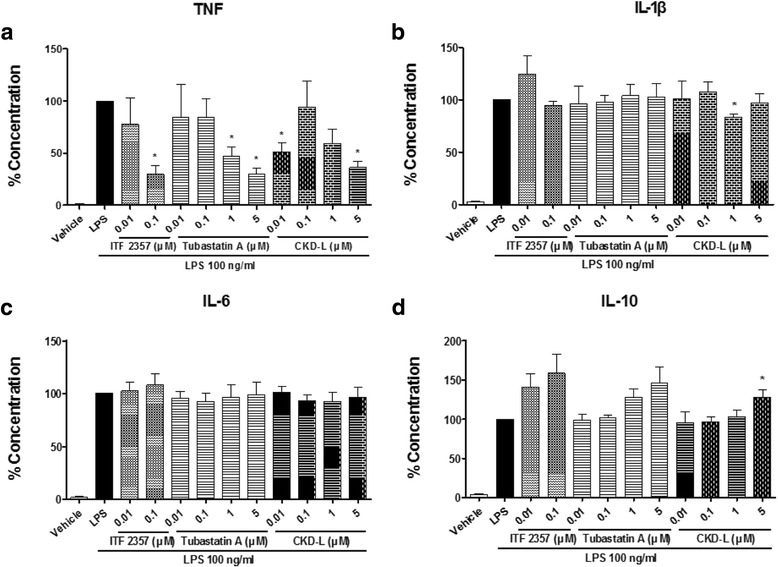
Effects of CKD-L on cytokine secretion in PBMC from RA patients (*n* = 5). PBMC from RA patients were cultured in a 48-well plate for 24 h in the presence of lipopolysaccharide (*LPS*; 100 ng/ml) and HDAC inhibitors at different concentrations (0.01 to 5 μM). Tumor necrosis factor (*TNF*) (**a**), IL-1β (**b**), IL-6 (**c**), and IL-10 (**d**) were measured in the culture supernatant by ELISA. *p < 0.01, vs LPS. *IL* interleukin

Isolated PBMC from RA patients were cultured with LPS (100 ng/ml) and HDAC inhibitors at different concentrations (0.01 to 5 μM) for 24 h. The secretions of TNF, IL-10, and IL-1β in the cell culture supernatant were measured by multiplex immunoassay, and IL-6 secretion in the cell culture supernatant was measured by ELISA. CKD-L significantly inhibited TNF at concentrations of 0.01 μM and 5 μM (Fig. 6a). Also, CKD-L inhibited IL-1β at a concentration of 1 μM (Fig. 6b) and increased IL-10 at a concentration of 5 μM (Fig. 6d). ITF 2357 inhibited TNF at a concentration of 0.1 μM, and Tubastatin A inhibited TNF at concentrations of 1 μM and 5 μM. ITF 2357 and Tubastatin A had no effect on IL-1β and IL-10. IL-6 production did not change following treatment with any HDAC inhibitor (Fig. 6c).

**Fig. 7:**
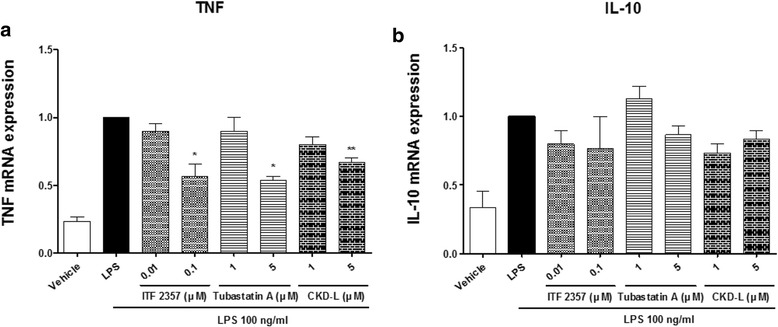
Effects of CKD-L on cytokine mRNA expression in PBMC from RA patients (*n* = 3). PBMC from RA patients were cultured in a 48-well plate for 24 h in the presence of lipopolysaccharide (*LPS*; 100 ng/ml) and HDAC inhibitors at different concentrations (0.01 to 5 μM). Total RNA was extracted from harvested cells and cDNA was synthesized by RT-PCR. Tumor necrosis factor (*TNF*) (**a**) and interleukin-10 (*IL-10*) (**b**) mRNA expression levels were analyzed by real-time PCR. *p < 0.001, **p < 0.01, vs LPS

Real-time PCR was conducted to measure the mRNA levels of TNF and IL-10. Total RNA was extracted from harvested cells and cDNA was synthesized by RT-PCR and then amplified. TNF mRNA expression was significantly decreased after treatment with a high concentration (5 μM) of CKD-L (*p* < 0.001) (Fig. 7). ITF 2357 and Tubastatin A also decreased TNF mRNA expression (*p* < 0.001). However, IL-10 mRNA levels did not change after treatment with CKD-L.

**Fig. 8:**
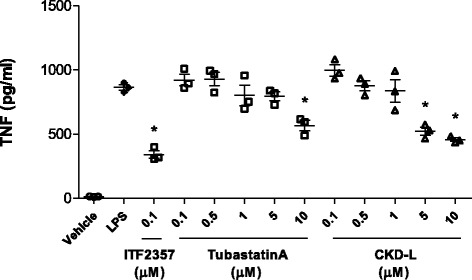
Tumor necrosis factor (*TNF*) secretion in PMA-activated THP-1 cells (*n* = 3). THP-1 cells (1 × 10^5^ cells/well) were cultured in a 24-well plate and activated with PMA (10 ng/ml) for 24 h to induce macrophage differentiation. PMA-activated THP-1 cells were treated with vehicle or HDAC inhibitors (0.1 to 10 μM) for 24 h and then treated with 100 ng/ml lipopolysaccharide (*LPS*) for 4 h. The secretion of TNF was measured by ELISA. *p < 0.001, vs LPS

THP-1 cells were activated with PMA for 24 h to induce macrophage differentiation. After differentiation, they were treated with vehicle or HDAC inhibitor (0.1 to 10 μM) for 24 h and then treated with 100 ng/ml LPS for 4 h. TNF was significantly decreased after treatment with CKD-L in a dose-dependent manner (*p* < 0.001) (Fig. 8). TNF secretion was also significantly inhibited by ITF 2357 (*p* < 0.001) and Tubastatin A (*p* < 0.001).

**Fig. 9:**
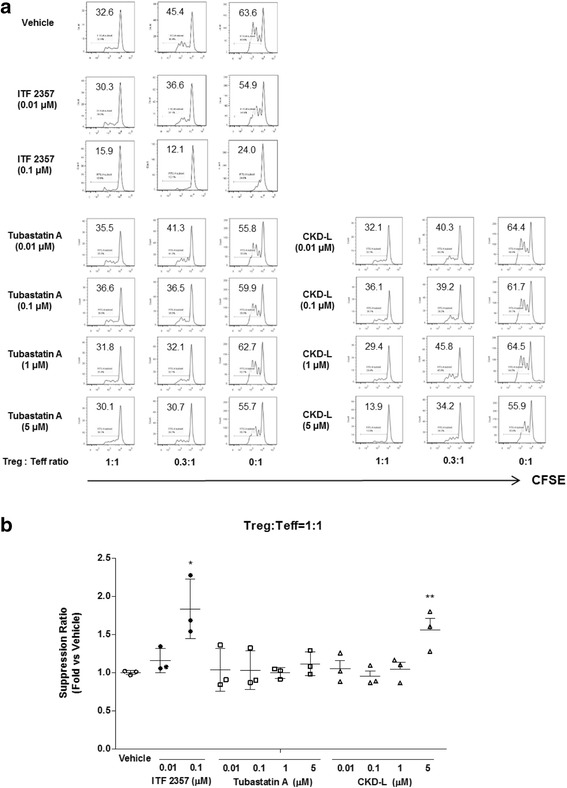
Effects of CKD-L in the suppression assay using induced regulatory T (*Treg*) cells and effector T (*Teff*) cells from RA patients (*n* = 3). Treg cells induced from RA patients were mixed with carboxyfluorescein succinimidyl ester (*CFSE*)-labeled Teff cells at ratios of 1:1, 0.3:1, and 0:1. The mixed cells were incubated with vehicle or HDAC inhibitors (0.01 to 5 μM) in the presence of Dynabeads® human T-activator CD3/C28 (one bead to four cells) for 3 days. **a** The proliferation of Teff cells was assessed by CFSE dilution using flow cytometry (representative data). **b** Suppression ratio (fold inhibition of cell proliferation by HDACi vs vehicle) at the ratio of Treg:Teff = 1:1. *p <0.001, **p < 0.05, vs vehicle

Induced Treg cells derived from RA patients were mixed with CFSE-labeled Teff cells at ratios of 1:1, 0.3:1, and 0:1. The mixed cells were incubated with vehicle or HDAC inhibitor (0.01 to 5 μM) in the presence of Dynabeads® human T-activator CD3/C28 (one bead to four cells) for 3 days. Proliferation of Teff cells was inhibited after treatment with CKD-L (5 μM) and ITF 2357 (0.1 μM) compared to vehicle (Fig. 9a). As the proportion of induced Treg cells was increased, proliferation of Teff cells was inhibited. In addition, in the Teff cell-only condition, ITF 2357 inhibited the proliferation of Teff cells but CKD-L did not have an effect. Therefore, the suppressive effect of CKD-L seems to be mediated by Treg cells. Tubastatin A had no suppressive effect in the suppression assay. The suppression ratio (fold versus vehicle) at 1:1 ratio of Treg:Teff was increased 1.5 times by CKD-L treatment and 1.8 times by ITF 2357 treatment compared to vehicle treatment (*p* < 0.05 and *p* < 0.01, respectively) (Fig. 9b).

## Discussion

Epigenetic regulation potentially influences the pathogenesis of RA and can provide therapeutic targets for the treatment of RA [35]. HDAC inhibitors that modulate the activities of HDAC and HAT have been reported to have potential anti-inflammatory effects on RA in many studies [5, 22–25]. In addition, HDAC inhibitors ameliorated joint inflammation and bone destruction in animal experiments, including in the CIA model [3, 5, 36]. Therefore, in the present study, we hypothesized that CKD-L could have beneficial effects on CIA. We found that CKD-L significantly decreased both the arthritis score and the histological score by blocking CIA progression.

We assessed the effect of CKD-L on the function of Treg cells. Treg cells and Teff cells were isolated from splenocytes of C57BL/6 mice and cocultured. Proliferation of Teff cells was inhibited after treatment with CKD-L or Tubastatin A in a dose-dependent manner. The suppression ratio (fold inhibition of cell proliferation by HDACi vs vehicle) was approximately two times greater after CKD-L treatment compared to vehicle treatment (data not shown).

In RA, activated CD4^+^ T cells have an important role in initiating and perpetuating chronic inflammation [37]. Based on their distinctive cytokine secretion profiles and functions, human CD4^+^ T cells can be divided into two major subtypes of cells, known as Th1 and Th2 cells. Th1 cells produce the proinflammatory cytokines IFN-γ, TNF, and IL-2, and promote macrophage activation, induce delayed-type hypersensitivity, and are involved in cell-mediated immunity. Th2 cells have been associated with downregulation of macrophage effector functions, they produce the anti-inflammatory cytokines IL-4, IL-5, IL-10, and IL-13, and mediate allergic immune responses [37–39]. IgG2a production is associated with a Th1 response, whereas IgG1 production is associated with a Th2 response [40]. Therefore, we hypothesized that CKD-L can increase or maintain the level of IgG1 and decrease the level of IgG2a in serum from animals with CIA. We measured the levels of serum IgG1 and IgG2a by ELISA. However, the levels of serum IgG1 and IgG2a did not change significantly after CKD-L treatment (data not shown).

HDAC inhibitors have been reported to reduce the levels of TNF, IL-1α, IL-1β, and IFN-γ in LPS-stimulated normal PBMC and reduce the levels of proinflammatory cytokines such as TNF and IL-6 in PBMC of RA patients [1, 24, 26, 28]. It was also reported that inhibition of HDAC3 suppresses the inflammatory gene expression, including type I IFN production in RA FLS [41]. We found that CKD-L inhibited the secretion of TNF and IL-1β, and increased the secretion of IL-10 in PBMC of RA patients treated with LPS and HDAC inhibitors. Also, as assessed by real-time PCR, CKD-L significantly inhibited TNF mRNA levels, in accordance with the results of measurements taken from the cell culture supernatant. Tubastatin A and ITF 2357 inhibited TNF secretion, but had no effect on IL-1β or IL-10. CKD-L, which regulates several cytokines such as TNF, IL-1β, and IL-10, is expected to play an important role in abnormal immune responses in RA patients.

In RA, T cells and macrophages are major sources of proinflammatory cytokines and the activation, survival, and apoptosis of these cells may be regulated by HDAC and HAT [27]. It is known that HDAC inhibitors suppress TNF and IL-6 production and transcription of cytokines in macrophages, and induce apoptosis in macrophages [28]. We found that CKD-L inhibited TNF secretion in PMA-activated THP-1 in a dose-dependent manner. Increased histone acetylation can regulate TNF production by controlling the access of transcription factors to promoter motifs directly or by regulating nucleosome remodeling.

Treg cells play an immunosuppressive role by producing TGF-β or inhibiting Teff cells [29]. Treg cells from RA patients are defective in the suppression of proinflammatory cytokine production [30]. TNF in RA decreases the immunosuppressive effect by inducing the malfunction of Treg cells. It is known that inhibition of HDAC6 enhances the suppressive activity of Treg cells in inflammation and autoimmunity [12]. In the present study, in C57BL/6 mice, CKD-L increased CTLA-4 expression in CD4^+^CD25^+^Foxp3^+^ T cells more effectively than did Tubastatin A, and inhibited the proliferation of Teff cells on suppression assay. In the suppression assay on PBMC from RA patients, we found that CKD-L significantly inhibited the proliferation of Teff cells compared to vehicle. As the ratio of Treg cells increased, the proliferation of Teff cells was inhibited to a greater degree. Interestingly, ITF 2357 inhibited the proliferation of Teff cells when only Teff cells were present in culture. On the other hand, CKD-L had no effect when only Teff cells were present. Therefore, the suppressive effect of CKD-L seems to be regulated through Treg cells. Tubastatin A had no suppressive effect in the suppression assay. These results suggest that CKD-L promotes the suppressive function of Treg cells. HDAC inhibitors can increase acetylation of histone and Foxp3, leading to increased expression of Foxp3, and they increase the production and suppressive function of Treg cells [42]. Foxp3 proteins in Treg cells are present in a dynamic protein complex containing HAT and HDAC enzymes, and Foxp3 itself is acetylated on lysine residues. In addition, it has been reported that CTLA-4 expression is associated with acquisition of a suppressive function in activated human CD4^+^CD25^–^ T cells [43].

There are few studies on the comparative effect of selective versus nonselective HDAC inhibitors. HDAC6 has been reported to modulate many important biological processes, including cell migration, immune responses, viral infections, and the degradation of misfolded proteins. These diverse functions of HDAC6 can be used as potential therapeutic targets in various diseases, such as systemic lupus erythematosus, cancer, and diabetes [10, 11]. Our data demonstrated that CKD-L, a selective HDAC6 inhibitor, decreased the arthritis score and protected against joint destruction in the CIA model, and reduced the expressions of TNF and IL-1β, and increased the expression of IL-10 in PBMC from RA patients. Also, CKD-L increased CTLA-4 expression and the suppressive function of Treg cells. Our data suggest that CKD-L may have a beneficial effect in the treatment of RA and further studies are needed to establish its role as a potential therapeutic agent.

## Conclusions

CKD-L, a selective HDAC6 inhibitor, decreased the arthritis score in CIA, reduced the expression of TNF and IL-1β, and increased the expression of IL-10 in PBMC from RA patients. CKD-L increased CTLA-4 expression and the suppressive function of Treg cells. These results suggest that CKD-L may have a beneficial effect in the treatment of rheumatoid arthritis.

## Abbreviations

ACR: American College of Rheumatology
BSA: Bovine serum albumin
CD: Cluster of differentiation
cDNA: Complementary DNA
CFA: Complete Freund’s adjuvant
CFSE: Carboxyfluorescein succinimidyl ester
CIA: Collagen-induced arthritis
CTLA: Cytotoxic T-lymphocyte antigen
DMARD: Disease-modifying antirheumatic drug
DMSO: Dimethyl sulfoxide
dNTP: Deoxynucleotide triphosphate
EDTA: Ethylenediaminetetraacetic acid
ELISA: Enzyme-linked immunosorbent assay
FBS: Fetal bovine serum
FLS: Fibroblast-like synoviocytes
Foxp3: Forkhead box P3
H&E: Hematoxylin and eosin
HAT: Histone acetyltransferase
HDAC: Histone deacetylase
HSP: Heat shock protein
IFA: Incomplete Freund’s adjuvant
IFN: Interferon
IL: Interleukin
LPS: Lipopolysaccharide
MACS: Magnetic cell sorting
MTT: Methylthiazol tetrazolium
MTX: Methotrexate
NAD: Nicotinamide adenine dinucleotide
NSAID: Nonsteroidal anti-inflammatory drug
P/S: Penicillin streptomycin
PBMC: Peripheral blood mononuclear cells
PBS: Phosphate-buffered saline
PMA: Phorbol 12-myristate 13-acetate
PRX: Peroxiredoxin
RA: Rheumatoid arthritis
RT: Room temperature
RT-PCR: Reverse-transcription polymerase chain reaction
Teff cell: Effector T cell
TGF: Transforming growth factor
TNF: Tumor necrosis factor
Treg cell: Regulatory T cell
